# Editorial: Angiogenesis and tumor metastasis

**DOI:** 10.3389/fonc.2022.1129736

**Published:** 2023-01-17

**Authors:** Qiang-Zhe Zhang, Yi-Pan Zhu, Michal A. Rahat, Julia Kzhyshkowska

**Affiliations:** ^1^ State Key Laboratory of Medicinal Chemical Biology and College of Pharmacy, Tianjin Key Laboratory of Molecular Drug Research, Nankai University, Tianjin, China; ^2^ Immunotherapy Laboratory, Carmel Medical Center, and the Ruth and Bruce Rappaport Faculty of Medicine, Technion-Israel Institute of Technology, Haifa, Israel; ^3^ Institute of Transfusion Medicine and Immunology, Mannheim Institue for Innate Immunosciences (MI3), Medical Faculty Mannheim, Heidelberg University, Mannheim, Germany; ^4^ German Red Cross Blood Service Baden-Württemberg – Hessen, Mannheim, Germany; ^5^ Laboratory for Translational Cellular and Molecular Biomedicine, Tomsk State University, Tomsk, Russia

**Keywords:** tumor metastasis, angiogenesis, EndoMT, EMT, biomarker

Angiogenesis, the process of forming new blood vessels from existing vasculature, allows the delivery of oxygen and nutrients to the tumor and supports its progression. Therefore, this necessary process has been recognized as one of the hallmarks of cancer (1). However, its role in promoting and sustaining metastasis is not yet fully elucidated. In cancer, the balance between pro- and anti-angiogenic factors is disrupted in favor of the former, leading to the activation of the “angiogenic switch.” Cytokines such as VEGF and its inhibitor TNFSF15 that are secreted from the cells within the tumor microenvironment (TME) participate in the regulation of angiogenesis (2). Different molecules, namely protein factors, exosomes, and non-coding RNAs, can mediate the effect on different cells within the TME and activate signaling pathways that are involved in the process of angiogenesis (3). The resulting new blood vessels are usually leaky and permeable, and they promote the infiltration of immune cells and the progression of the tumor. On the other hand, they can also provide a pathway for the metastatic tumor cells to escape from the primary tumor and reach the circulation, thus supporting metastasis.

The term “metastasis” refers to the migration of cancer cells from their primary site to other organs in the body. This process requires that the metastatic cells become invasive through intravasation into a lymphatic or blood vessel, survive in the circulation, and then extravasate into the distant organ (4). These migratory and invasive properties require that the epithelial-to-mesenchymal transition (EMT) process be activated. This allows the metastatic cancer cells to disseminate and enter blood vessels recently formed by angiogenesis. Once the cancer cell is lodged in the new environment, it must undergo the opposite process of mesenchymal-to-epithelial transition (MET) in order to reacquire its ability to proliferate and colonize the distant sites, which is necessary to form a metastatic, secondary tumor in the distant organ (5). Another important transition of cells’ states is the endothelial-to-mesenchymal transition (EndoMT), which contributes to both metastatic extravasation and intravasation. During EndoMT, vasculature endothelial cells (ECs) lose their endothelial cell-cell junctions, allowing the transendothelial migration of metastatic cells. Once metastatic colonization has been achieved, further steps are needed to start a new round of angiogenesis to nourish the new tumor. This Research Topic provides an updated overview of new regulatory mechanisms and potential targets in tumor angiogenesis and metastasis.

Two papers suggest new mechanisms involved in inducing angiogenesis. Gao et al. found that STAT6, which is activated by IL-13, can bind to the neuropilin-1 (NRP1) promoter and increase NRP1 expression in ECs, thus promoting tumor angiogenesis. STAT6 inhibitor (AS1517499) and STAT6 siRNA could suppress tumor angiogenesis and growth in tumor xenograft models, suggesting that STAT6 may be a potential target for anti-angiogenesis therapy. Park et al. demonstrated that radiation stimulated angiogenesis in Glioblastoma multiforme (GBM) *via* the growth/differentiation factor-15 (GDF15), which in turn fosters the crosstalk between ECs and GBM cells. The radiation-induced secretion of GDF15 from ECs activated the VEGFA promoter in glioma cells through the pMAPK1/SP1 signaling pathway, consequently promoting angiogenesis in GBM. Silencing GDF15 ameliorated radiation-induced VEGFA upregulation in glioma cells and increased the angiogenic activity of ECs. Therefore, GDF15 is a potential target for radiation-induced angiogenesis in GBM patients receiving radio-chemotherapy.

Increased migration and invasion of tumor cells are necessary for tumor metastasis. Xue et al. found that mesenchymal stem cell-transformed cancer-associated fibroblasts (MTCAFs) secreted more ICAM-1 that could bind the LFA-1 receptors in colon cancer cells, activate Akt and STAT3 signaling, and promote the migration, invasion, and metastasis of the colon cancer cells, both *in vitro* and in xenograft tumor models. Tyumentseva et al. revealed that the chemotherapeutic agent dacarbazine changed the transcriptomic profiling of melanoma cells and affected primarily genes related to movement, migration, invasion, and adhesion pathways. Specifically, the upregulation of plexin A2 (PLXNA2) reduced the migratory and invasive capabilities of melanoma cells, establishing PLXNA2 as a new marker for the invasion and migration of melanoma cells.

Bai et al. reviewed how tumor-derived exosomes (TDEs) promote tumor metastasis by reshaping the TME and the distant metastatic niche. By delivering non-coding RNAs and proteins to tumor cells, TDEs promote the EMT program and activate ECM-degrading proteases to enhance tumor cell migration. TDEs can promote angiogenesis by activating macrophages and ECs. Intravasating tumor cells are protected by the layer of TDEs and by the ability of TDEs to suppress circulating NK, T, and B cells. When extravasating, TDEs prepare the pre-metastatic niche, and their integrins determine organotropism. In brain metastasis, Geng et al. demonstrated that exosomes derived from highly metastatic non-small cell lung cancer (NSCLC) cells are internalized in brain microvascular ECs and release the lncRNA LINC01356 that targets junctional proteins such as claudin-5, N-cadherin, Occludin, and ZO-1. This leads to the disruption of the blood-brain barrier (BBB) and promotes brain metastasis in lung cancer.

Presented next are three papers that describe a new mechanism of osteosarcoma metastasis. Lee et al. implicated CXCL1, which enhances the expression and nuclear translocation of NF-kB through the CXCR2/FAK/PI3K/AKT pathway, resulting in the upregulation of VCAM-1. This cascade enhances the migration, invasion, and metastatic ability of osteosarcoma cells to the lung. Shao et al. found that tetraspanin-9 (Tspan9) can promote EMT and lung metastasis of osteosarcoma cells and also interact with integrin β1 and enhance the FAK-RAS-ERK1/2 signaling cascade in osteosarcoma cells. The finding that the downregulation of Tspan9 could inhibit lung metastasis in a mouse model of osteosarcoma suggests that Tspan9 may be used as a new therapeutic target in osteosarcoma. Interestingly, Kuriyama et al. reported that the pigment epithelium-derived factor (PEDF) increased osteosarcoma cells’ extravasation to the kidneys and lungs by triggering MET and inhibiting EndoMT through the PEDF-laminin receptor axis. Collectively, these three studies highlight new mechanisms of osteosarcoma metastasis and organotropism.

The search for useful and reliable biomarkers to determine prognosis and the response to immunotherapy in cancer patients is ongoing. Wang et al. implemented bioinformatics analyses to determine a risk score based on the identification of 31 key, differentially expressed angiogenesis-related genes (DE-ARG) in GBM patients. This signature proved to be effective in predicting the survival rate and the response to immunotherapy and useful in providing a personalized program for GBM patients. Similarly, Li et al. identified an angiogenesis score based on a pan-cancer analysis across 33 human cancer types. Higher values of the score were correlated with infiltration of macrophages and inducible Tregs, and patients with lower score values had a favorable prognosis and better responses to immunotherapy. Dong et al. identified another potential biomarker, the macrophage-related secreted phosphoprotein 1 (SPP1), for early lymph node metastasis in lung adenocarcinoma. LncRNA AC037441, which was negatively associated with SPP1 and infiltrating macrophages, was also implicated in the regulation of early lymph node metastasis. Jiang et al. constructed a scoring model based on the expression of 4 RNA-binding proteins to predict the risk for peritoneal metastasis in gastric cancer. The model could excellently predict TNM stage and prognosis, and the generated nomograms could help in making clinical decisions. Collectively, these studies suggest that data mining could accelerate the evolution of cancer therapy in the era of big data.

Myeloid-derived cells are key components of TME. Stefanescu et al. found that TGFβ/TGFβR2 signaling in myeloid cells could differently regulate metastasis in tissue-specific ways, depending on the specific TME. TGFβ signaling in myeloid-derived cells promoted lung metastasis of Lewis lung carcinoma cells by suppressing the activity of CD8+ T cells, while the same signaling pathway increased liver metastasis of colon cancer cells by enhancing M2-monocytic polarization. An interesting case of pancreatic solid pseudopapillary neoplasm (SPN) with peritoneal and hepatic metastasis is illustrated by Wang et al. The identification of the mutation variants in the PTEN and CTNNB1 genes was used to stabilize the disease with the targeted drugs sunitinib and everolimus. This report demonstrates how molecular studies can help explain the mechanism of SPN occurrence and contribute to the decision-making process for precision treatments.

Collectively, this series of articles highlights the importance of angiogenesis in tumor metastasis, sheds light on new mechanisms that promote metastasis, and suggests new therapeutic targets to decrease angiogenesis and the metastatic cascade in the treatment of cancer.

## Author contributions

All authors listed have made a substantial, direct, and intellectual contribution to the work and approved it for publication.
