# Erratum: Editorial: Angiogenesis and tumor metastasis

**DOI:** 10.3389/fonc.2024.1364928

**Published:** 2024-01-12

**Authors:** Frontiers Production Office

**Affiliations:** Frontiers Media SA, Lausanne, Switzerland

**Keywords:** tumor metastasis, angiogenesis, EndoMT, EMT, biomarker

Due to a production error, there was a mistake in the funding statement as published. “This work was supported by the state contract of the Ministry of Science and Higher Education of the Russian Federation “Genetic and epigenetic editing of tumor cells and microenvironment in order to block metastasis” o. 075-15-2021-1073 and by Tomsk State University Development Programme (Priority 20-30).”

The correct funding statement appears below. The publisher apologizes for this mistake.

This work was supported by the National Natural Science Foundation of China (No. 82073233), Israel Science Foundation (ISF) grant number 2607/20, the state contract of the Ministry of Science and Higher Education of the Russian Federation “Genetic and epigenetic editing of tumor cells and microenvironment in order to block metastasis” o. 075-15-2021-1073 and Tomsk State University Development Programme (Priority 20-30).

The original version of this article has been updated.

